# Awareness among mothers on acute respiratory tract infections in under five children at north Gujarat, India

**DOI:** 10.6026/97320630019713

**Published:** 2023-06-30

**Authors:** Mahalakshmi B, Bharatbhai Patel Nidhi, Sivasubramanian N, Ramalakshmi G, Krishnan A, Navinbhai Patel Divyankakumari

**Affiliations:** Nootan College of Nursing, Sankalchand Patel University, Visnagar, Gujarat -384315, India; College of Nursing, S.G.R.R University, Dehradun, Uttarkhand-248001,India

**Keywords:** Acute respiratory infections, awareness, mothers, under five children

## Abstract

Acute respiratory infections (ARI) account for 14.3% of new born mortality and 15.9% of death among children aged 1 to 5 in India, making them significant contributors to morbidity and mortality in children. However, the bulk of these fatalities would be avoided if mothers were aware of the symptoms and signs of infections so that prompt referrals could be made. The goal of the current study was to gauge mothers of children under the age of five's knowledge of ARI and examine how well an education programme did in raising that knowledge. The information was gathered from 60 mothers of young children under the age of five in different areas around Gujarat State in India. The pre-intervention-post-test methodology was used. The mean pre-test observation score was 8.51, the mean post-test score was 19.31, and the mean difference was 11. There were 60 samples; the pre-test and post-test scores had standard deviations of 4.59 and 2.54, respectively. The calculated "t" value was also 17.29, the DF was 59, and the table value of "t" was 1.67. These findings demonstrate that the educational programme on ARI was successful in increasing the mother's knowledge.

## Background:

In India, acute respiratory infections (ARIs) are the main cause of mortality in children under the age of five. Every child experiences five episodes of ARI on average per year in poor nations, which account for 30%-50% of all paediatric outpatient visits and 20%-30% of pediatric admission [1]. The magnitude of ARI was shown to rise when the literacy rate fell. Health education can alter the attitudes and practices of parents and other family members toward obtaining medical care and caring for the ARI child at home [2].India has a serious public health issues with childhood ARI/pneumonia, despite the lack of reliable epidemiological data on its occurrence. In India, mortality from pneumonia accounts for almost one-fourth of all deaths in children under the age of five. Children who are exposed to solid fuel consumption, early newborns, malnourished children, and children who are not exclusively breastfed have a higher chance of developing pneumonia than other children [3]. A KAP (knowledge, attitude, and practice) survey was given to a random sample of 140 women with 265 children from the registered families of the Urban Health Training Centre at Aligarh in India, in order to determine the relationship between mothers' literacy levels and their comprehension of ARIs. It was discovered that 58 mothers had full understanding of ARI management, 61 had partial awareness, and 21 either had no information or gave unsatisfactory answers. Only 15.5% of the 40 illiterate mothers possessed full knowledge, compared to 75% of the 40 literate mothers. This research emphasises the value of health education initiatives in raising awareness [4]. ARIs are significant contributors to child morbidity and mortality in India, where they contribute for 15.9% of mortality among children aged 1 to 5 and 14.3% of infant mortality. However, the bulk of these fatalities would be avoided if mothers were aware of the symptoms and signs of infections so that prompt referrals could be made. Additionally, mothers must offer their kids comfort care when they are ill[5].In order to learn how mothers in a rural location diagnose pneumonia in children, what treatments they use for mild acute respiratory diseases and pneumonias, and the feeding techniques they use, 156 mothers were questioned. More than half of the mothers chose not to treat their children for minor ARIs episodes or to simply utilize natural therapies [6]. An epidemiological investigation of acute respiratory infections (ARI) in an urban area revealed that mothers were frequently unable to distinguish between mild and severe infections. Inappropriate action was the outcome of poor diagnostic skills that were exacerbated by a lack of understanding of the proper management of various types or degrees of ARIs. As a result, there were many people who self-medicated and few people who used health services [7]. Therefore it is of interest to assess the awareness of mothers of fewer than five children regarding ARI and to evaluate the effect of education program in boosting awareness of mothers.

## Methodology:

Experimental research design was used for the current investigation. The study's objective was to assess the effectiveness of an education programme in raising mothers of children under the age of five awareness of ARI and how to manage it. 60 mothers of children under the age of five provided the data. Purposive sampling technique was used to select samples from several villages in Gujarat, Mehsana District. The existing awareness was assessed using a self-structured questionnaire. After pre intervention test, the mothers were provided with educational pamphlets and teaching program with the help of pictures and charts. The identification of sign and symptoms, importance of utilizing health care services were included in the education program. The effectiveness of the education program was assessed seven days after the start of the education program. The collected data was analyzed by various statistical methods such as mean, standard deviation and chi-square.

## Results:

50% of mothers were in the age group of 31-35 years, 41.66% mothers had higher education, majority 93.33% of mothers were housewives and 60% of the family earning a monthly income between Rs.10,000 to 15,000. 50% of mothers had 2 children, 58.33% mothers were from joint family and majority 66.66% mothers had no education regarding respiratory tract infections.

**Figure 1:**
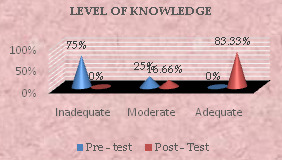
Diagram showing percentage distribution of the sample according to the pre-test and post-test Level of knowledge

Figure 1 shows that in the pre-test conducted before to the implementation of the educational programme, 75% of the sample had insufficient knowledge, 25% had intermediate knowledge, and none had adequate knowledge. In the post-test, the sample's knowledge had significantly improved, with 16.66% gaining intermediate knowledge, 83.33% gaining acceptable knowledge, and nobody having inadequate knowledge.

Table 1 shows the comparison between pretest and posttest observation score regarding knowledge of under-five mothers regarding acute respiratory tract infection in under five-year children. The mean pre-test observation score was 8.51 and the mean post test score was the 19.31, and the mean difference was 11. Number of sample was 60, and the Standard Deviation was 4.59 in pre-test and 2.54 in post-test score, also the calculated "t" value was 17.29 and the DF =59, and the table value of 't' was 1.67. This results shows that the educational awareness program on ARI was effective in boosting the mother's knowledge.

## Discussion:

The goal of the current study was to gauge mothers of children under the age of five knowledge of ARI and examine how well an education programme did in raising that knowledge. The study findings indicate that mothers of children under the age of five knew too little about ARI and how to manage it. A descriptive cross-sectional study carried out in 16 randomly chosen clusters across two districts in the Indian state of Maharashtra supports these findings. In the chosen clusters, all mothers with children under the age of five were included. Mothers were found to have poor feeding habits, poor hand cleanliness, and inadequate awareness of the symptoms and signs of pneumonia [8]. Another study was done to investigate mothers' knowledge, attitudes, and practises regarding ARI as well as their health-seeking behaviour. A descriptive study was conducted on 204 mothers using pre-test semi-structured pro-forma, and data on awareness of ARI, attitudes toward seeing a doctor, and practises surrounding antibiotic use were gathered. Self-medication was viewed as being used by 52.5% of people [9].The present study also aimed to assess the effectiveness of teaching programs about ARI in children under the age of five in raising awareness among mothers of young children. The study conclusions demonstrated how well-received teaching programmes were raising mothers' awareness of ARI in young infants. A comparable study was carried out to determine whether mothers' knowledge, attitudes, and practices had changed as a result of receiving health information on respiratory illnesses in kids. Compared to mothers of children for whom sessions were not done, moms of children for whom educational sessions were conducted had considerably improved knowledge, attitude, and practices [10 -see PDF].The mothers of children under the age of five in the semi-urban area of Sasaram participated in a study to evaluate the efficiency of a structured training program about knowledge on acute respiratory tract infections. The structured teaching program used in this study was successful in educating mothers of children under five about acute respiratory tract infections [11-see PDF]. These research evidences supports the fact that educational programs are helpful in creating and improving awareness among mothers of under five years children regarding ARI.

## Conclusion:

The current study sought to measure mothers of children under the age of five on knowledge of ARI and analysis the importance of education program in raising awareness of the condition. According to the results of this study, the educational program on ARI was successful in increasing the mother's knowledge. The mothers' literacy rate had a favourable impact on their awareness of ARI in young children.

## Figures and Tables

**Table 1:** Mean, Standard Deviation, Mean difference, and "t" value of knowledge scores obtained before and after teaching program

**Parameter**	**Mean**	**Standard deviation**	**Mean difference**	**'t' value**	**Table 't' value**	**DF**
Pre-test	8.51	4.59
Post-test	19.31	2.54	11	17.29	1.67	59
